# Influence of reward learning on visual attention and eye movements in a naturalistic environment: A virtual reality study

**DOI:** 10.1371/journal.pone.0207990

**Published:** 2018-12-05

**Authors:** Alexia Bourgeois, Emmanuel Badier, Naem Baron, Fabien Carruzzo, Patrik Vuilleumier

**Affiliations:** 1 Neuroscience Department, Laboratory for Behavioral Neurology and Imaging of Cognition, University of Geneva, Geneva, Switzerland; 2 Swiss Center for Affective Sciences, University of Geneva-CISA, Geneva, Switzerland; University of Plymouth, UNITED KINGDOM

## Abstract

Rewards constitute crucial signals that motivate approach behavior and facilitate the perceptual processing of objects associated with favorable outcomes in past encounters. Reward-related influences on perception and attention have been reliably observed in studies where a reward is paired with a unidimensional low-level visual feature, such as the color or orientation of a line in visual search tasks. However, our environment is drastically different and composed of multidimensional and changing visual features, encountered in complex and dynamic scenes. Here, we designed an immersive virtual reality (VR) experiment using a 4-frame CAVE system to investigate the impact of rewards on attentional orienting and gaze patterns in a naturalistic and ecological environment. Forty-one healthy participants explored a virtual forest and responded to targets appearing on either the left or right side of their path. To test for reward-induced biases in spatial orienting, targets on one side were associated with high reward, whereas those on the opposite side were paired with a low reward. Eye-movements recording showed that left-side high rewards led to subsequent increase of eye gaze fixations towards this side of the path, but no such asymmetry was found after exposure to right-sided high rewards. A milder spatial bias was also observed after left-side high rewards during subsequent exploration of a virtual castle yard, but not during route turn choices along the forest path. Our results indicate that reward-related influences on attention and behavior may be better learned in left than right space, in line with a right hemisphere dominance, and could generalize to another environment to some extent, but not to spatial choices in another decision task, suggesting some domain- or context-specificity. This proof-of-concept study also outlines the advantages and the possible drawbacks of the use of the 3D CAVE immersive platform for VR in neuroscience.

## Introduction

Rewards represent crucial signals that induce approach behaviour, promote learning, and determine various choices, and that are essential for survival. Theories of approach behaviour and motivation have proposed that reward signals encoded in the midbrain dopaminergic regions may prime perceptual and attentional mechanisms mediated by cortical systems, and thus cause reward-associated stimuli to become more salient and attention-drawing [1]. Experimental evidence of reward-related influences on attention shows that reward modulates visual search performance, either speeding up the detection of previously rewarded targets [2] or diverting attention to previously rewarded distractors [3–8]. Likewise, several studies found that reward may determine oculomotor salience during visual exploration by biasing not only perceptual mechanisms but also the saccadic eye-movement system [9–12]. Importantly, the impact of reward on visual representations seems to occur independently of the more strategic establishment of attentional set as determined by explicit or implicit goal formation [13–15], suggesting that these effects are driven by distinct motivational signals evoked by particular sensory stimuli.

Remarkably, many previous studies investigating these effects used psychophysical experimental paradigms in which reward was paired with a unidimensional low-level visual feature, such as the color, shape, or orientation of simple objects (e.g. bars, letters). Real-life environments, however, are composed of multidimensional and changing visual features. Interestingly, one recent study used photographs of real-world scenes to investigate the impact of reward on perception [16]. They demonstrated that reward could influence attention at the level of semantic categories, composed of visually heterogeneous objects. A few other studies examined how reward could induce spatial biases in attention towards locations in space associated with a positive reinforcement regardless of the targets’ sensory features [17, 18]. These findings suggest that reward history might induce plastic changes at the level of priority maps of space, in addition to low-level visual cortices. Yet, to our knowledge, no studies have investigated how reward guides human perception and spatial attention in more complex naturalistic environments, where subjects have to freely explore a dynamically changing visual scene.

Here we employed a 3D CAVE immersive platform for virtual reality (VR) to investigate the impact of rewards on attentional orienting and gaze patterns with an ecological experimental design. Behavioral responses and eye-movements were recorded while participants explored a virtual forest in which they had to detect animals appearing on either their left or right side. Animal targets (birds and rabbits) on one side were associated with high reward, whereas those on the opposite side were paired with a low reward.

Our first aim was to test whether asymmetric reward history would lead to asymmetric performance in target detection and eye movement patterns, in order to confirm and extend the notion that reward learning can bias attention to spatial locations [13, 17, 18] in addition to elementary visual features [5, 8]. Our second aim was to determine whether value-driven attentional capture is context-specific or transfers to another context. Della Libera and Chelazzi [7] previously reported that attentional capture by a previously rewarded visual feature could be observed several days after the reward association learning phase and generalized to new tasks and new stimuli relative to those used during learning [19]. Interestingly, however, Anderson et al. [20] demonstrated that a reward-related but task-irrelevant color distractor could either capture or not capture attention depending on its contextual history (i.e., only when re-occurring in the same scene), suggesting instead that value-associated learning might be context-specific. In the current study, we compared how reward-related biases acquired in a given environment would transfer to another environment with distinct visual features and distinct spatial demands (i.e. exploring a virtual castle rather than a forest). Thirdly, we also tested whether value-related influences could generalize to spatial behavior in other non-perceptual choices. A recent study demonstrated that subjects are more likely to choose options that they fixated longer, even if those options were previously rated as less appealing [21]. Likewise, computational models demonstrated that combining information about value and perceptual saliency can better account for choice behavior than models based on a rational comparison of options values only [22, 23]. We therefore hypothesized that reward-related biases in spatial attention and saccadic eye-movements might in turn lead to biases in subsequent spatial choices in the same environment (i.e. selecting route trifurcations in the virtual forest).

By using VR, we could design a novel paradigm allowing us to manipulate various visual features and task parameters in a naturalistic manner, and thus test for different facets of reward-induced effects on human behavior.

## Materials and methods

### Participants

Forty-one healthy participants took part in this study. Nineteen participants (mean age 25 years, range 20–35, 10 males) were presented with high rewards in left space, while another group of 22 participants (mean age 29 years, range 18–37, 10 males) received high rewards in right space. All participants were right-handed and had normal or corrected-to-normal vision and no history of neurological or psychiatric disorders. This study was approved by the Neurosciences Cliniques Ethics Committee of the University Hospital of Geneva (HUG; no 09–316). Written informed consent was obtained for each participant before participation and adhered to the principles detailed in the Declaration of Helsinki.

### Apparatus

An advanced 3D CAVE immersive platform (BBL Immersive System, see http://bbl.unige.ch/researchmodules/bbl-is/te/ for a complete description of the apparatus) was used to run the experiment. The 3D CAVE system comprises four screens, arranged in a cube shaped layout. Visual 3D rendering with depth perception was obtained using active stereoscopic glasses, coupled with online control of the visual scene. Seven video projectors (Digital Projections, TITAN QUAD 3D WUXGA) were used to project high-resolution images (1920*1200 pixels) at 120 frames per second. Projection was performed on the four acrylic coated screens (DaLite, 2.8 m wide and 2.4 m high) with a high contrast ratio and brightness (1600 cd/m^2^ per screen). An optical motion tracking system composed of eight infrared cameras (Vicon, Bonita 3) was used to capture participant movements. Head movement was tracked using a set of reflective markers fixed on the active stereoscopic glasses. The experiment was programmed using Unity 3D software and a custom software toolkit (Geneva Virtual Reality Elements, GeVRE). Eye movements were recorded for all participants throughout the experiment using a 10 Hz SMI eye-tracking goggle system. Finally, a joystick held with the right hand was used to navigate through the visual environment.

### Stimuli and experimental procedure

Participants were required to explore a virtual forest and two castles located inside the forest. They were instructed that they had two goals: to detect target animals in the forest, and to explore the courtyard of the two castles to find a key, in order to escape the castle.

**Fig 1:**
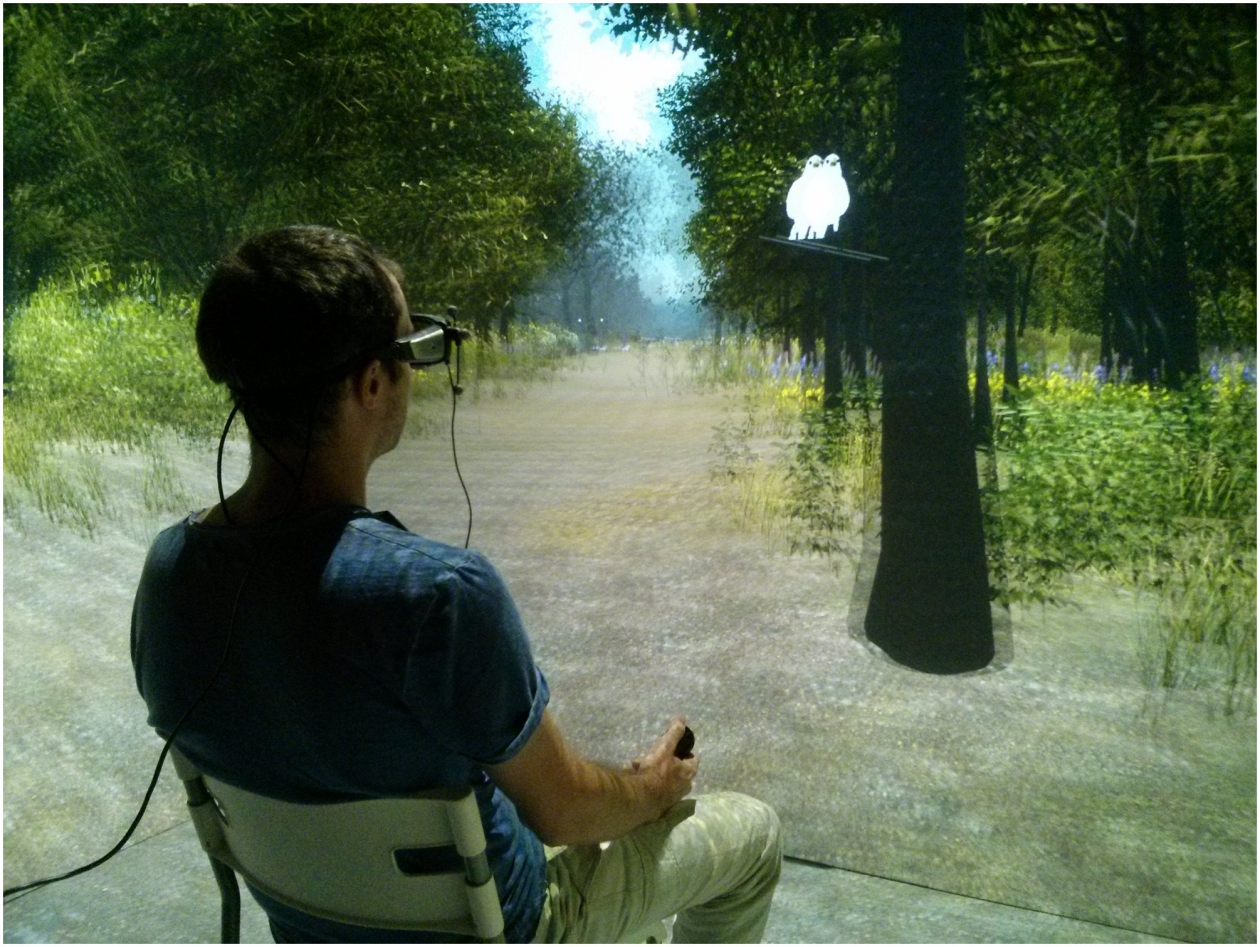
Illustration of the experimental setup.

**Fig 2:**
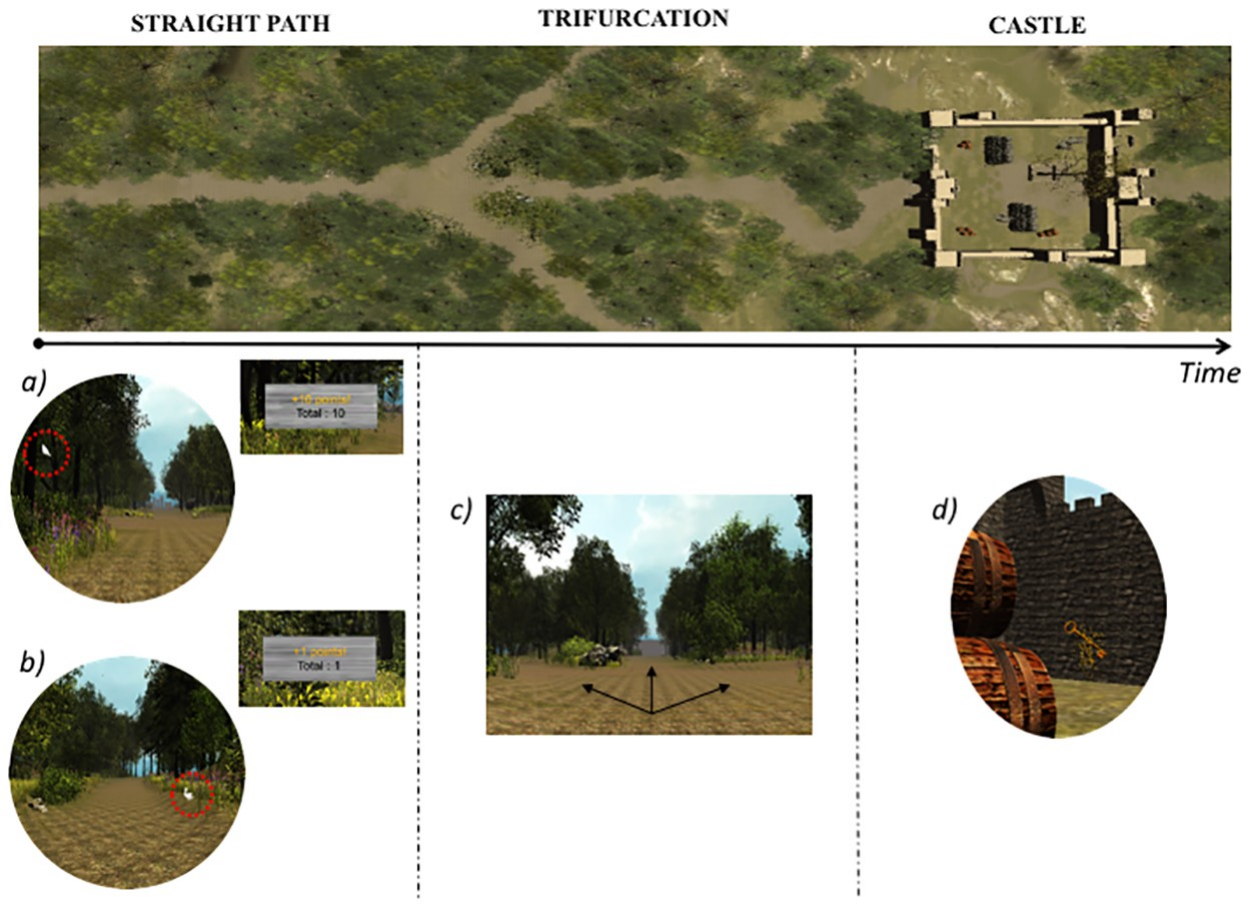
Illustration of the timeline (top of the figure) and snapshot bullets depicting target events (bottom of the figure) during the VR experiment. Participants had to explore a forest by walking along a path and find animals (rabbits or birds) that could appear on either the left or the right side with equal likelihood. Correct detection of animals on the left (Group Left_HR, left hemispace highly rewarded) or on the right side of space (Group Right_HR, right hemispace highly rewarded) was associated with a high reward (10 points), whereas correct detection of animals in the opposite hemispace was associated with a low reward (1 point) (**snapshot** bullets *a)* and *b)*). During this exploration phase, participants were also occasionally faced with path trifurcations (**snapshot** *c)*) and asked to choose to either go straight, turn 45 degrees to the left, or turn 45 degrees to the right. The forest was divided into three main sections (A, B, C), with a castle courtyard being displayed at the end of sections A and C. Participants were required to explore this courtyard and to find a hidden key (**snapshot** *d)*) (located in the opposite corner relative to the entrance in both castles). The second castle was presented with a 180 degrees rotation of its walls and internal layout.

The forest was composed of three main sections (A, B, and C). In each section, participants had to walk along a path and to detect the appearance of 6 white rabbits sitting on the ground (lower visual field) and 6 white birds perched on nearby trees (upper visual field). Animals were equally distributed on the left and on the right side of the path, corresponding to the left and right hemispace of participants. To induce spatial biases in attention [17, 18], the correct detection of animals either on the left side (Group Left_HR, left hemispace highly rewarded) or on the right side of space (Group Right_HR, right hemispace highly rewarded) was associated with a high reward (10 points), whereas correct detection of animals on the opposite hemispace was associated with a low reward (1 point). After each successful detection, a scoreboard appeared at the location of the correctly detected animal to inform the participant about the points earned on that trial, as well as the total reward accumulated across all trials. Participants were asked to detect as many animals as possible and to maximize their total number of points. Importantly, participants were not informed of the reward contingencies. No feedback was given when participants missed an animal. Participants were not provided monetary compensation based on performance (i.e. points were not translated to subsequent monetary payment at the end of the experiment). The forest was globally symmetric on both sides of the path and made of trees with gently moving branches and leaves, accompanied by an ongoing soft rustling sound. Participants could not make a U-turn during their exploration of the forest and could not leave the main path displayed in the middle of the visual scene (Figs 1 and 2, snapshot bullets *a)* and *b)*).

In addition, within each main section of the forest, participants were occasionally presented with a trifurcation and asked to choose one out of three possible directions in order to continue their exploration: either going straight, turning 45 degrees to the left, or turning 45 degrees to the right. Each path was perceptually identical. Each section comprised 6 trifurcations, with two animals displayed in each subsection (Fig 2, snapshot bullets *c)*).

**Fig 3:**
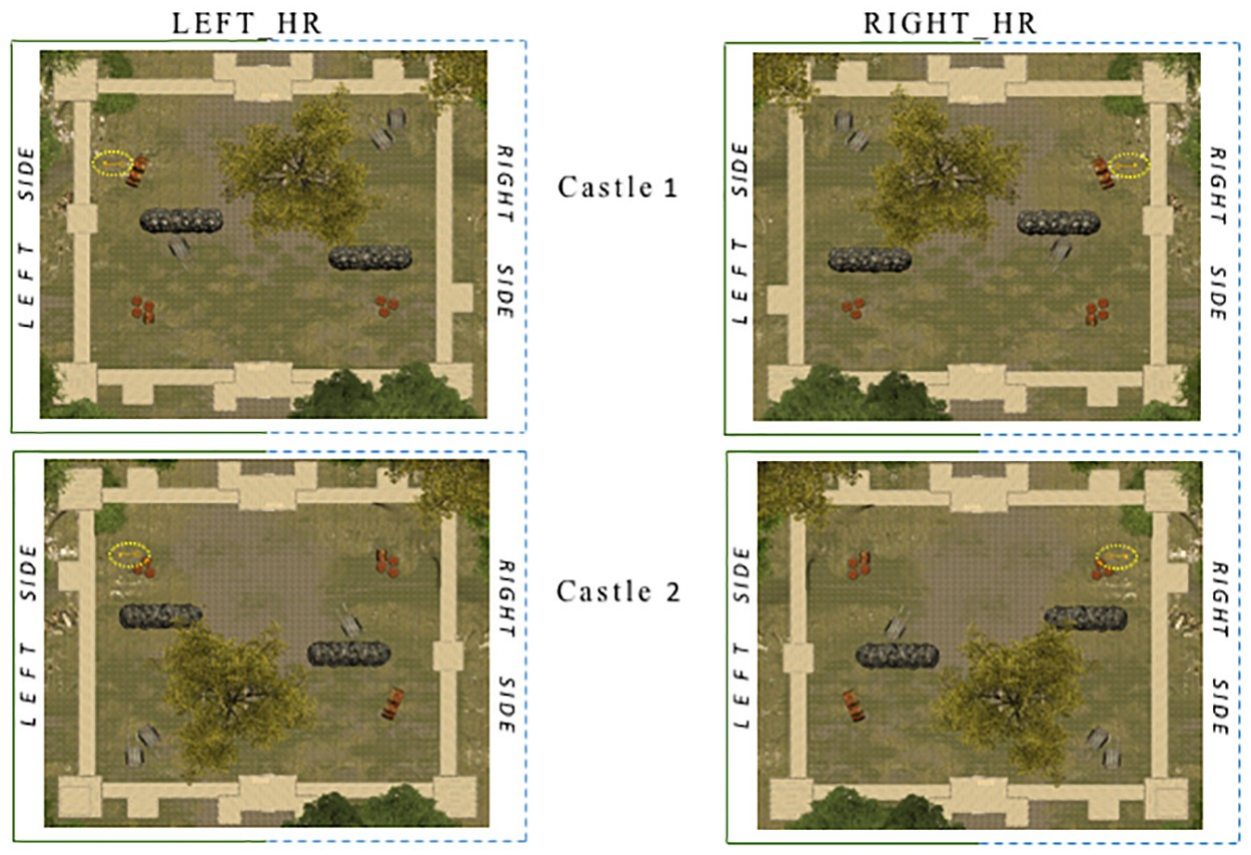
Illustration of the courtyard layout for the two castles (Castle 1 at the end of section A, Castle 2 at the end of section C), for the Left_HR group (left panel) and for the Right_HR group (right panel). The key-target (surrounded by a yellow circle) was hidden at an unknown location, in the left opposite corner relative to the entrance for the Left_HR group and in the right opposite corner for the Right_HR group. The second castle was presented with a 180 degrees rotation. The key-target was presented in the same location in each castle.

Finally, at the end of the sections A and C, participants arrived at a castle and had to enter through its door into a large courtyard. The castle was not visible from the decision points. Upon entrance through the door, the perceived visual layout of the courtyard was entirely symmetrical. At this point, they were required to explore the courtyard and to find a key, hidden at an unknown location (i.e. in the left opposite corner relative to the entrance for the Left_HR group and in the right opposite corner for the Right_HR group). The second castle at the end of the section C was presented with a 180 degrees rotation. After participants found the key, another door opened in the wall. Participants could then exit the castle and continue their exploration in the next section of the forest (Fig 2, snapshot bullets *d)* and Fig 3).

Throughout the experiment, participants sat on a chair 1.5 meters from the front screen. The experiment started with a training session, followed by the experimental phase. Participants could control their navigation in the virtual environment using the joystick as well as the head-tracking system. In order to minimize the influence of large body or head movements, we instructed participants to maintain a position generally looking straight ahead, with the path displayed in front of them, and to avoid turning around. Gaze fixation points were defined in coordinates anchored on the VR scene (rather than in terms of eye- or head-centered coordinates as typically done for eye movement studies with fixed scenes presented on a computer screen). By integrating eye position data with head position data, the location of gaze position towards the right or left side of the scene was calculated relative to the VR space. In order to improve realism, the auditory environment (humming of bird songs and tree leaves) was diffused through four surrounding speakers during the whole experiment.

After the end of the experiment, a systematic debriefing was obtained using 3 successive questions to probe if participants had guessed any association between asymmetries in target locations and reward delivery. We started with a very general question in order to avoid any bias in the response, by asking “Did you notice something during the experiment that allowed you to win more or less points?”. If the answer was negative, we further specified the question by asking “Do you think that animals could give you more or less points?”. If the answer was still negative, we asked a final question: “Some participants reported that animals on the left (or right as a function of the group of participants considered) gave more points. What is your opinion about this statement?”. If the answers to these 3 questions were negative, participants were considered *unaware* of the association between reward and asymmetries in target locations. Otherwise, they were included in the group of participants that were *aware* of the association between reward and asymmetries in target locations.

In order to assess reward-related influences on visual exploration in the VR forest, we computed the mean number of correct target detections (pooling rabbits and birds), as well as the number of eye-gaze fixations made in the high-rewarded hemispace (left in Left_HR group, right in Right_HR group) and the opposite low-rewarded hemispace over time (i.e. for sections A, B, and C in the forest). Then, in order to test whether value-driven attentional orienting is context-specific or transfers to another context, we examined the spontaneous orientation and navigation path during exploration of the castle courtyard, in both the middle (i.e. end of the section A) and final part (i.e. end of the section C) of the experiment. Finally, in order to investigate if any reward-induced biases acquired during exploration could influence other spatial behavioral choices, we analyzed the number of decisions to go straight or turn left or right when arriving to trifurcations along the path in the forest.

## Results

### Target detection hits and eye-movements analysis

Three subjects in the Right_HR group were discarded from the analysis because of feelings of nausea caused by VR settings, which caused them to discontinue the experiment. We first performed a repeated-measure analysis of variance (ANOVA) on the mean number of correct target detections, with the within-participant factors of Target side (left hemispace, right hemispace) and Section in the forest (A, B, C), as well as the between-participant factor of Group (Left_HR, left hemispace highly rewarded; Right_HR, right hemispace highly rewarded). Planned paired *t*-tests were used for post-hoc comparisons.

**Fig 4:**
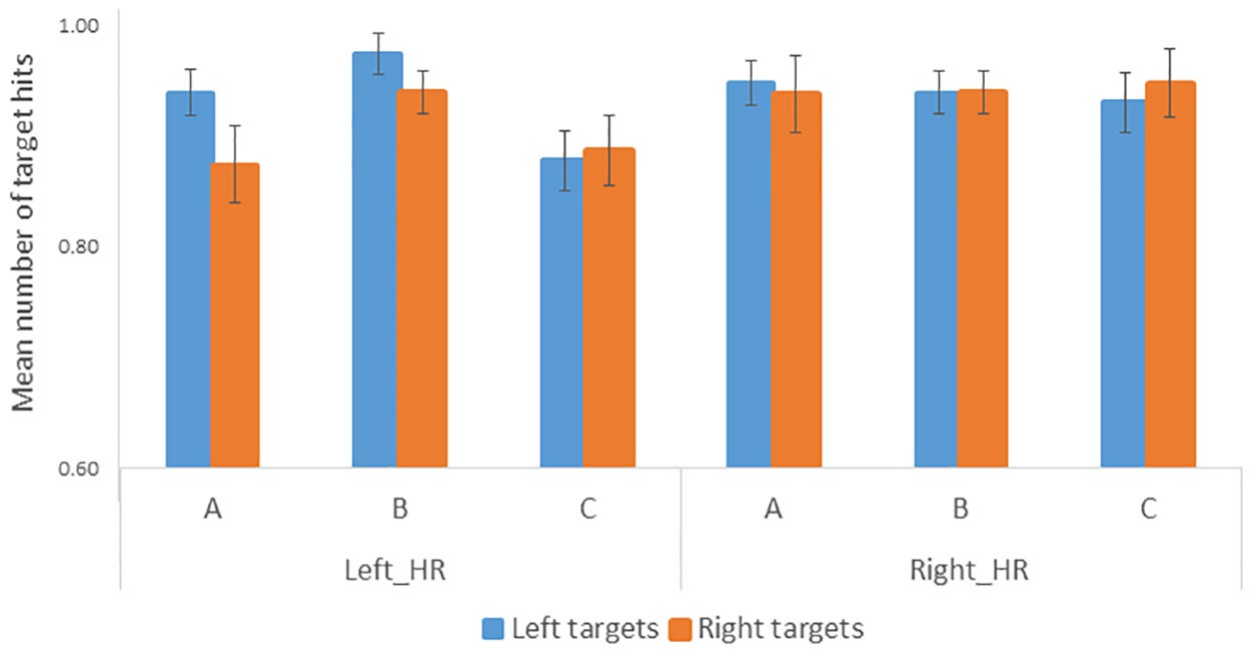
Mean number of correct detections for left and right animal targets during sections A, B, and C for Left_HR participants (correct detection of animals on the left associated with a high reward) and Right_HR participants (correct detection of animals on the right associated with a high reward).

Target detection performance was generally close to ceiling given that targets were highly visible, and accuracy was therefore similarly high across all sections. Statistical analysis indeed indicated no significant main effects or interactions (all *p*s > .099; Fig 4).

We then asked whether reward could influence oculomotor exploration patterns, and compared the total number of gaze points directed towards the left / high-rewarded space versus right / low-rewarded space during the sections A, B, and C. To reliably distinguish eye-movements unambiguously directed to one or the other side, we discarded gaze points located in a region extending between 20 degrees to the left and 20 degrees to the right of an imaginary line going through the midline of the path. In addition, one Left_HR subject was discarded from this analysis due to technical problems during eye-movements recording. A repeated-measure ANOVA was performed on the total number of gaze points recorded during each exploration phase, with the within-participant factors of Side (left, right) and Section (A, B, C), and the between-participants of Group (Left_HR, Right_HR). This analysis revealed significant main effects of Side, F(1,35) = 8.36, MSE = 56065, *p* = .007, Section, F(2,70) = 53.07, MSE = 31008, *p* < .001, and a significant interaction between Side and Section, F(2,70) = 8.82, MSE = 8962, *p* < .001. Participants showed more frequent eye-gaze fixations in the left hemispace compared to the right hemispace, especially during section B and C (section A, mean number of left gaze points, 828, right gaze points, 808, t(36) = .52, *p* = .63; section B, mean number of left gaze points, 638, right gaze points, 491, t(36) = 4.18, *p* < .001; section C, mean number of left gaze points, 609, right gaze points, 500, t(36) = 3.26, *p* = .002). The interaction between Side, Section and Group was not significant, F(2,70) = .50, MSE = 8962, *p* = .61.

**Fig 5:**
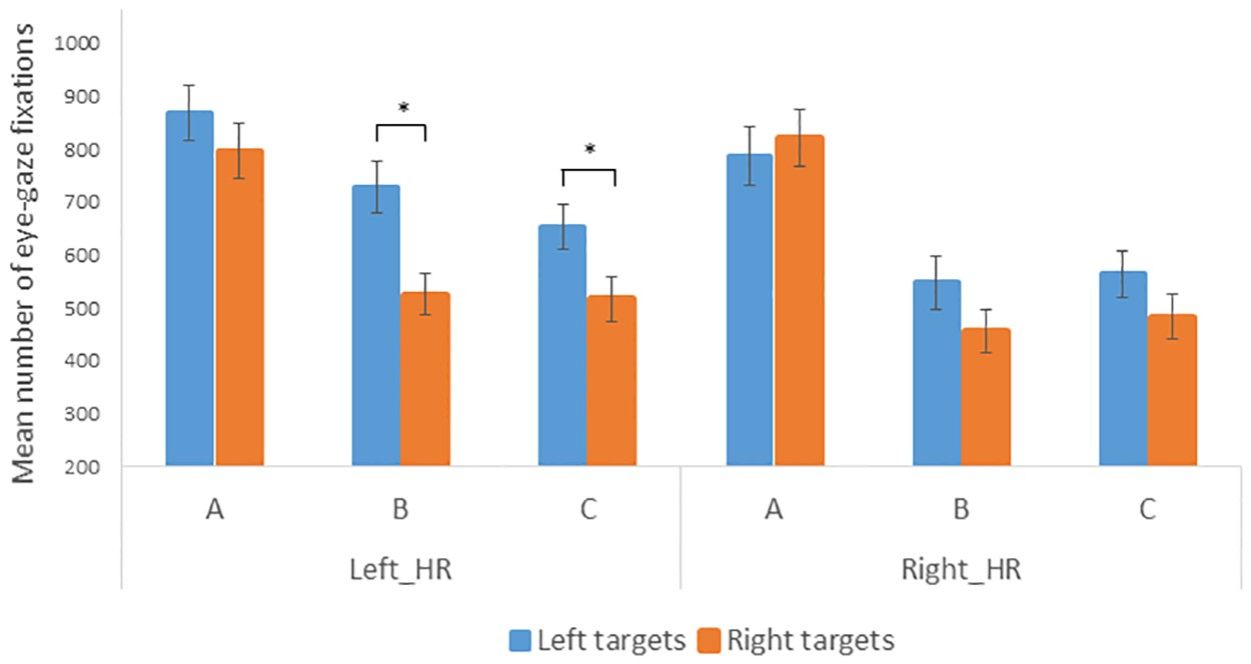
Mean number of gaze points directed towards the left / high-rewarded and the right / low-rewarded hemispace during sections A, B, and C for Left_HR participants (correct detection of animals on the left associated with a high reward) and Right_HR participants (correct detection of animals on the right associated with a high reward).

However, given our primary hypothesis of an influence of reward learning on eye-movements, planned comparisons were performed between eye-movements performed towards the left versus the right hemispace across the three sections, and for participants receiving high rewards on the left (Left_HR) or the right side of space (Right_HR). Whereas no difference between the two sides of space was present during section A in both groups (Left_HR: mean number of left gaze points, 868, right gaze points, 796, t(18)<1; Right_HR: mean number of left gaze points, 787, right gaze points, 820, t(17)<1), Left_HR participants made significantly more eye-movements towards the left than the right during section B (mean number of left gaze points, 729, right gaze points, 526, t(18) = 2.98, *p* = .008). This was also the section with the highest number of target detection hits (Fig 4). The same pattern of results was observed during section C (mean number of left gaze points, 653, right gaze points, 517, t(18) = 2.48, *p* = .023). In sharp contrast, no bias was observed for Right_HR participants during sections B and C (section B, mean number of left gaze points, 548, right gaze points, 456, t(17) = 1.80, *p* = .090; section C, mean number of left gaze points, 564, right gaze points, 483, t(17) = 1.32, *p* = .21) (Fig 5 and Table 1).

**Table 1:** Total number of gaze points directed towards the left / high-rewarded and the right / low-rewarded hemispace, as well as towards the central space (not taken into account in the analysis) during sections A, B, and C for Left_HR participants (correct detection of animals on the left associated with a high reward) and Right_HR participants (correct detection of animals on the right associated with a high reward).

	Section A		
	Left hemispace	Central space	Right hemispace
Left_HR	15636	37263	14150
Right_HR	14161	35196	14757
	Section B		
	Left hemispace	Central space	Right hemispace
Left_HR	13245	27396	9470
Right_HR	9864	26091	8213
	Section C		
	Left hemispace	Central space	Right hemispace
Left_HR	11843	27521	9334
Right_HR	10156	27424	8688

Interestingly, these effects arose even though reward asymmetry was entirely irrelevant to the target side probability (which might affect detection performance accuracy), given that animal targets appeared equally on either side. Moreover, during debriefing, only two out of 38 final participants guessed that the different amounts of reward were associated with a specific side of the path. The pattern of results was identical when these two participants’ data were removed.

### Castle exploration

In order to determine whether any spatial biases in attentional orienting induced by asymmetric reward history would transfer to another context, we studied the strategy of spatial exploration when participants entered the castle’s courtyard, which was displayed at the end of the sections A and C (Fig 3). Unlike the visual search for animals in the forest, exploration inside the courtyard was free, that is, participants could turn and go in all possible directions, and no visible target was presented upon entrance. To assess spatial behavior, we computed a heatmap of exploration trajectory using the virtual position of the participant over time with a frame rate recording of 10 Hz. This analysis was performed from the time participants entered the castle until the time they found the hidden key and could exit the courtyard. The number of visited positions in the heatmap was then submitted to a repeated measures ANOVA with the within-participant factors of Side (left vs right part of the courtyard) and Castle episode (first, second), and the between-participant factor of Group (Left_HR, left hemispace highly rewarded; Right_HR, right hemispace highly rewarded).

**Fig 6:**
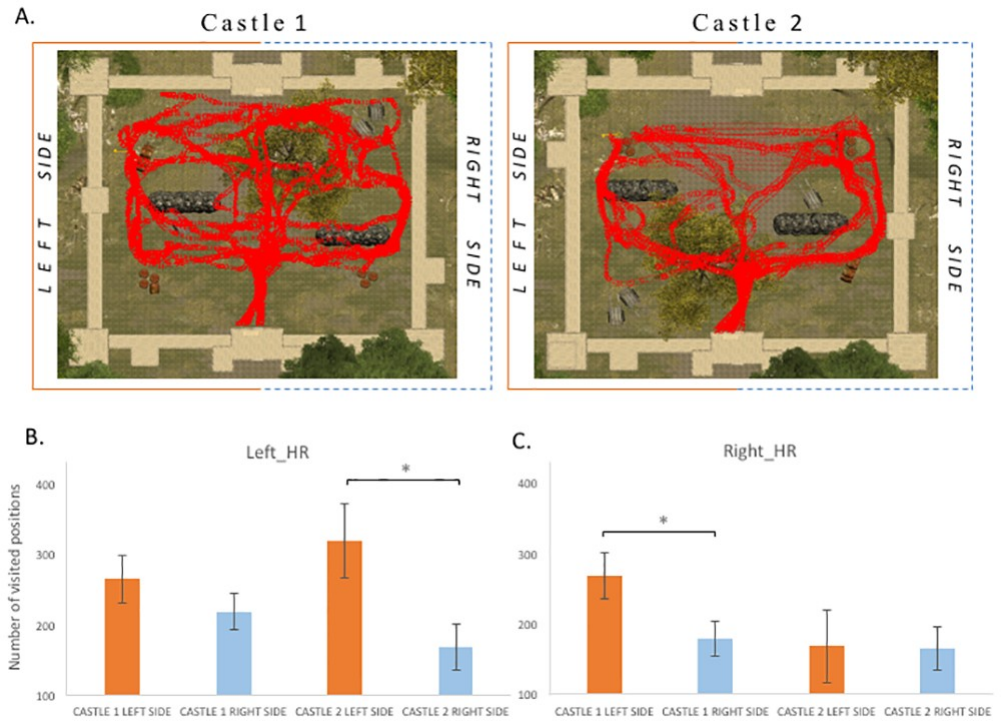
**A**. Illustrative heatmap depicting exploration trajectories of all Left_HR participants during their search in the courtyard, from their entrance until they found the hidden key. **B**. Group results for Left_HR, mean number of visited positions in the left and right parts of the courtyard in each castle. **C**. Group results for Right_HR, mean number of visited positions in the left and right parts of the courtyard in each castle.

Results from this analysis indicated a significant main effect of Side, F(1,35) = 6.00, MSE = 32580, *p* = .0019, with significantly more frequently visited positions in the left part of the courtyard compared to the right side. The interaction between Side, Castle episode, and Group was also marginally significant, F(1,35) = 3.90, MSE = 21423, *p* = .056. Following our hypothesis and guided by the eye-movement results, we performed planned comparisons between conditions, which indicated that whereas no difference was observed during the first castle episode for Left_HR participants (number of left visited positions, 265, vs right positions, 217, t(17) = 1.14, *p* = .27), during the second castle episode this group demonstrated a left sided spatial bias with more visited positions in the left than the right side of the courtyard (number of left visited positions, 319, vs right positions, 168, t(17) = 2.11, *p* = .050). In contrast, Right_HR participants demonstrated a left sided spatial bias during the first castle episode (number of left visited positions, 267, vs right positions, 178, t(17) = 2.21, *p* = .040), which was not present during the second castle episode (number of left visited positions, 167, vs right positions, 164, t(17)<1) (Fig 6). These data point to a possible but modest asymmetry in exploration developing in different directions across the two groups during this phase.

### Path direction analysis

Finally, in order to test for any reward-related influences on spatial choices beyond attentional orienting, we compared the number of decisions on path trifurcations where participants could go straight or turn left or right (towards the high/low-rewarded hemispace depending on group), during each of the three forest sections (A, B, and C), for Left_HR and Right_HR participants separately. For both groups, Chi-square tests for independence revealed that reward-related asymmetries did not generalize to these spatial behavioral choices (Left_HR, Chi-square = 6.25, df = 4, *p* = .18; Right_HR, Chi-square = 7.84, df = 4, *p* = .097) (Table 2).

**Table 2:** Total number of decisions on path trifurcations to take a straight forward direction or to turn left or right, during each of the three forest sections (A, B, and C) for Left_HR and Right_HR participants.

Group	Directions	Sections		
		A	B	C
*Left_HR*	Straight forward	51	40	58
	Left	29	35	29
	Right	33	39	27
*Right_HR*	Straight forward	57	44	63
	Left	27	29	17
	Right	39	30	22

## Discussion

We used a 3D CAVE immersive platform for VR to investigate how motivational signals occurring due to reward learning could influence human spatial behavior in naturalistic conditions. Our results demonstrate that reward-associated stimuli can effectively modulate visual exploration and produce automatic biases in eye-movements, favoring search in the side of space where previous targets were highly rewarded relative to the less rewarded side. However, this effect occurred only following rewards paired with left-sided stimuli, not following rewards on the right side. Thus, when correct detection of left targets led to higher rewards, participants made more frequent eye movements towards the left (high rewarded) side. These effects arose even though reward asymmetry was entirely irrelevant to the target probability, and despite unawareness of such asymmetry in all but two participants.

Our results accord with previous work revealing that strong biases can be induced by rewards on attentional and perceptual mechanisms [2, 3, 5, 6, 18, 24] but also on the saccadic eye-movement system [9–12, 25]. Interestingly, the impact of reward on attention and space representation most often occurs without explicit knowledge of the acquired value of visual cues, as also reported by several studies using visual search in laboratory settings [3, 5, 6, 12, 24, 26] and verified here by debriefing questions after our experiment.

In all previous studies, however, reward-related influences were observed by manipulating low-level visual features, such as color or shape, in highly arbitrary experimental conditions (visual search among basic shapes, such as line or symbols (but see [16, 27]). Instead, real life environments are composed of multidimensional visual features with complex objects and changing contexts. Thus, our new results extend this previous work by confirming that reward biases can operate on spatial representations in addition to elementary visual cues [17, 18] or specific object categories [27], and also by demonstrating that these effects can guide visuo-motor behavior in ecological, complex, and meaningful real-world scenes. These findings further support the notion that motivational signals induced by reward learning provide a valuable window on mechanisms that regulate visual exploration abilities in naturalistic situations, which might be exploited for various practical applications, including rehabilitation interventions in patients suffering from attentional disorders (such as neglect syndrome after stroke). Subsequent spatial biases in exploration occurred only when higher rewards were received on the left (not right) side, suggesting that the underlying brain mechanisms might be asymmetrically organized across the two hemispheres [28].

Converging evidence suggests that reward-related signals originate in midbrain dopaminergic regions, which may then modulate interactions between basal ganglia and sensory cortices [8, 29], as well as oculomotor regions such as the superior colliculus [30] or the frontal eye-field [31]. These effects may ultimately cause reward-associated features or spatial locations to become more salient and thus gain higher competition weights in attention priority maps that guide both overt and covert attentional orienting [1, 4, 18]. However, the asymmetry we observed here for reward–induced biases in eye-movements (arising for stimuli located in the left but not right hemispace) points to functional differences between the two hemispheres regarding neural circuits linking reward processing with spatial attention and oculomotor control. These findings add to previous work demonstrating asymmetries in dopaminergic responses in the human striatum which could then determine individual spatial biases during reward learning [32, 33]. Furthermore, recent neuroimaging investigations with PET found that the degree of right lateralization of dopaminergic release was predictive of subsequent attentional capture by reward–related stimuli in a visual search task [34, 35]. Taken together, these data suggest brain asymmetries that might support better reward learning in the right hemisphere and in turn lead to a stronger impact on attention orienting towards the left hemifield, in line with our results. Alternatively, compelling evidence has demonstrated a right-hemispheric dominance of attention-related processes in humans [36–41], including the well-established right pseudo-neglect phenomenon in healthy subjects [37, 42] and the classic left spatial neglect syndrome after right hemispheric stroke, characterized by a failure to take into account information coming from the left side of space [38, 43–45]. This right-hemispheric attentional dominance potentially also might account for (or contribute to) the fact that reinforcement learning processes produce differential effects on orienting systems across the two hemispheres, with greater modulation of right-sided networks and stronger subsequent attentional biases towards targets in the left hemispace [37, 46].

Reward in this study was not translated into monetary compensation, but relied on intrinsic motivation factors that made participants willing to obtain points without monetary gain. This could have contributed to asymmetries observed in behavior, given that the right hemisphere is also dominant in a number of affective and motivational processes [35, 47], but in addition could potentially weaken the reward effects observed in our paradigm. Future studies should examine the impact of stronger or direct reward feedback during learning, for instance by providing monetary compensation based on performance. In any case, our newly developed VR setup provides a valuable paradigm to assess the effect of various changes in the attention and eye-movement systems, for example after drug manipulation or in patient populations with either dysfunction in the dopaminergic pathways or damage to neural networks mediating visuo-spatial representations.

A second aim of our study was to determine whether value-driven attentional biases learned in a given environment could transfer to another task and environment. This was examined by testing spatial behavior in a new environment in VR (the castle phase) after participants were exposed to asymmetric reward in the first phase of their exploration (the forest phase). Whereas previous studies reported a full transfer of reward-associated effects to new tasks and new stimuli relative to those used during learning [7, 19], our results revealed only limited and time-dependent transfer of the spatial asymmetry induced by reward-association learning in the forest, to subsequent visuo-spatial exploration in the new castle environment. Thus, participants exposed to higher rewards on the left side of the VR path not only showed more frequent leftward eye-movement in the forest but also visited left-sided locations more often in the second castle yard, but not in the first castle yard. Moreover, this effect occurred specifically after left-sided rewards, not after right-sided rewards. These data suggest that some generalization might take place but requires time to develop. Other studies found that reward-associated but task-irrelevant color-based distractors can either capture or not capture attention depending on whether these features were previously rewarded in the same or in a different scene context [20]. Such transfer of reward-induced biases [5, 7] was reported in task conditions where reward was associated with particular visual objects or elementary features (e.g. color), whereas our experiment tested for generalization of spatial representations across different exploration tasks and different spatial layouts, rather than different objects. Taken together, these data suggest that reward influences on spatial attention might be sensitive to contextual information that modulates the access to and/or impacts reward associations held in memory, and might operate differently at different levels of visual-spatial processing.

Finally, we also tested if value-related influences could bias spatial choices beyond attentional orienting. Previous studies demonstrated an active role for gazing in preference formation [21, 48]. For instance, Vaidya et al. [21] showed that subjects were more likely to choose pieces of artwork if they previously fixated them for longer, even if these options were initially rated as less appealing. In our study we found that eye-movements were preferentially directed towards the left high-rewarded space during visual search in the VR forest, rather than to the right low-rewarded space. We therefore tested whether this acquired asymmetry in attention also generalized to spatial decisions at trifurcation points along the path in the forest. Indeed, one might hypothesize that reward learning induced plastic changes within priority maps of space, which may not only guide attention and eye movements but also render the high-rewarded hemispace more salient when choosing new directions to explore. However, our results indicated that reward history did not influence these behavioral choices. This could reflect different reward effects on spatial representations engaged across the different kinds of tasks (i.e. implicit guidance of attention versus explicit decision making; graded versus categorical spatial codes for exploration and path direction, respectively). Alternatively, reward history in this experiment may not have generated a sufficiently strong signal to influence spatial decisions at path trifurcation points.

The different spatial domains explored in this study may tap into different neural mechanisms, which could explain some of the dissociations observed. For instance, the search for the key-target during the castle exploration may require more strategic control, including the avoidance of re-visiting previously explored locations [38, 40, 41, 49, 50]. Moreover, these dissociations also might be due, at least partly, to differences in the time range inherent to each measure (eye-gaze fixations, visited locations in the castle, decisions for the turn-choices). This study also points out some limitations regarding the use of the VR. Indeed, the ecological but complex nature of this setting might decrease the specificity and sensitivity of the reward manipulation on attention, notably because of the presence of multimodal distracting information, higher task demands to master navigation in the environment, as well as occasional motion sickness (feelings of nausea) induced by the VR in a subset of participants. In addition, the role of other parameters enhancing the motivational value of rewards (e.g. monetary outcomes versus abstract points) must be further clarified. Thus, more research is necessary to examine the psychometric properties of this useful research tool compared to traditional computerized experimental apparatus.

To conclude, we exploited the richness and naturalistic complexity of a 3D CAVE immersive platform for virtual reality to investigate the impact of reward on attentional orienting and gaze patterns, and the range of such effects across different spatial behaviors. Our results demonstrate that reward-association can selectively bias the orienting of eye-movements in space, suggesting a modulation of saliency or priority maps by reward history. Reward-related influences, however, appear to be asymmetrically acquired (during initial learning) or expressed (during subsequent exploration), as they were observed only after reward associated with left-side targets, pointing to some right hemispheric dominance in this process. Furthermore, leftward biases might partly, but modestly, generalize to spatial exploration in another VR environment, but not to more categorical, non-perceptual spatial choices, suggesting some domain- or system-specificity.

This proof-of concept VR study highlights the possibility of examining complex spatial behavior in naturalistic settings, and shows both the benefits and possible limits of this approach. Thus, this work opens new perspectives to better understand and measure the impact of reward on vision and attention in ecological and meaningful real-world scenes.
